# Overview of the Importance of Biotics in Gut Barrier Integrity

**DOI:** 10.3390/ijms23052896

**Published:** 2022-03-07

**Authors:** Aleksandra Maria Kocot, Elżbieta Jarocka-Cyrta, Natalia Drabińska

**Affiliations:** 1 Department of Immunology and Food Microbiology, Institute of Animal Reproduction and Food Research, Polish Academy of Sciences in Olsztyn, Tuwima 10, 10-748 Olsztyn, Poland; a.kocot@pan.olsztyn.pl; 2 Department of Pediatrics, Gastroenterology and Nutrition, School of Medicine, Collegium Medicum University of Warmia and Mazury, Regional Specialized Children’s Hospital, Żołnierska St. 18A, 10-561 Olsztyn, Poland; elzbieta.jarocka@uwm.edu.pl; 3 Department of Chemistry and Biodynamics of Food, Institute of Animal Reproduction and Food Research of Polish Academy of Sciences, Tuwima 10, 10-748 Olsztyn, Poland

**Keywords:** gut barrier, leaky gut, intestinal permeability, probiotics, prebiotics, synbiotics, microbiota

## Abstract

Increased gut permeability is suggested to be involved in the pathogenesis of a growing number of disorders. The altered intestinal barrier and the subsequent translocation of bacteria or bacterial products into the internal milieu of the human body induce the inflammatory state. Gut microbiota maintains intestinal epithelium integrity. Since dysbiosis contributes to increased gut permeability, the interventions that change the gut microbiota and correct dysbiosis are suggested to also restore intestinal barrier function. In this review, the current knowledge on the role of biotics (probiotics, prebiotics, synbiotics and postbiotics) in maintaining the intestinal barrier function is summarized. The potential outcome of the results from in vitro and animal studies is presented, and the need for further well-designed randomized clinical trials is highlighted. Moreover, we indicate the need to understand the mechanisms by which biotics regulate the function of the intestinal barrier. This review is concluded with the future direction and requirement of studies involving biotics and gut barrier.

## 1. Introduction

Intestinal inflammatory diseases have become a real challenge in recent times. Chronic inflammation of different intensity is observed in inflammatory bowel diseases (IBD), overweight, obesity, type I diabetes (T1D), cardiovascular and neurodegenerative diseases, depression and anxiety [1]. These diseases have a varied aetiology; however, one of the factors potentially linking them is the increased permeability of the intestinal barrier [2]. It is shown that immune activation induces gut barrier dysfunction. A growing body of epidemiological, experimental and clinical data supports the close link between increased intestinal permeability and gastrointestinal as well as systemic disorders. The intestinal barrier is the first line of defence against pathogens and food antigens [3]. In a healthy organism, the intestinal barrier is characterized by selective permeability, which means that water, ions and low-molecular substances can freely pass through it, while it is impermeable to macromolecules, toxins, food allergens and pathogens [2]. When these substances leave the intestine, the immune system is over-activated, resulting in the induction of inflammation, which, if it persists for a long time, leads to serious health consequences [3]. Therefore, maintaining the integrity of the intestinal barrier is one of the most important aspects of ensuring health.

**Figure 1:**
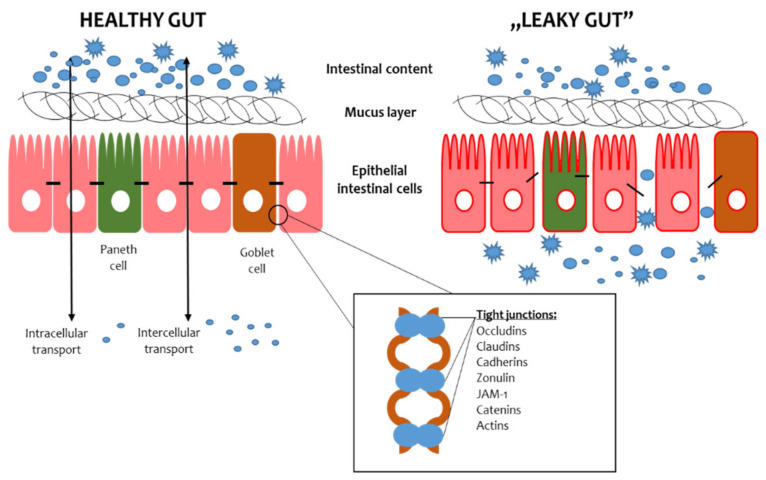
Scheme of the gut barrier structure. JAM-1—Junctional Adhesion Molecule 1.

The intestinal barrier consists of the mucus layer, intestinal microbiota, intestinal epithelial cells (IECs) and lamina propria (Figure 1) [4]. In the context of maintaining the integrity of the intestinal barrier, intestinal epithelial cells and microbiota are essential. The IECs provide a physical barrier to prevent substances from leaking out of the intestinal lumen [5]. The paracellular space between enterocytes is sealed by tight junctions (TJs), adherens junctions (AJs) and desmosomes [6]. The AJs and desmosomes adhere directly to enterocytes, conditioning their integrity, while TJs are found between the side parts of enterocytes. The TJs consist of transmembrane proteins, i.e., claudins, occludins, peripheral membrane proteins, i.e., zonula occludens (ZO), and regulatory proteins [6]. The microbiota is the most unstable element of the intestinal barrier since its composition depends on many factors, such as diet, lifestyle and medications [7]. The role of the microbiota in preserving the intestinal barrier largely depends on the fact that commensal microbes adhere to the intestinal mucosa and constitute an additional protective layer. In addition, intestinal bacteria, by taking up space on the surface of the IECs, prevent the adhesion of pathogens, thus protecting the IECs from their invasion and damage [8]. Furthermore, intestinal bacteria produce antimicrobial substances that inhibit the multiplication of pathogens and secrete mucous substances that create a dense environment in which the translocation of substances is difficult [9]. Thus, maintaining the integrity of the intestinal barrier requires cooperation between all its components.

The integrity of the gut barrier can be evaluated in several ways. On the one hand, there are functional tests, which are based on the assumption that different substances can or cannot cross the barrier unless it is compromised. The most commonly used functional test of this type is a sugar absorption test (SAT) [10]. This non-invasive test measures the absorption and urinary excretion of two non-digestible sugars following oral administration. The lactulose–mannitol (LM) test is one of the most commonly used in vivo permeability assays. Lactulose excretion reflects the abnormal intestinal permeability of large molecules due to compromised TJs, while mannitol excretion indicates a decreased epithelial surface area [10]. In healthy conditions, more mannitol than lactulose should be absorbed in the gut. The results of SAT are usually presented as a ratio of lactulose to mannitol [11]. The “multi sugar test” is based on the administration of sucrose, lactulose, sucralose, erythritol and rhamnose simultaneously to assess gastro-duodenal, small intestinal and large intestinal permeability. Moreover, other substances such as polyethylene glycols (PEG) 400 Da and ^51^chromium-ethylenediaminetetraacetic acid (^51^Cr-EDTA) are used [12]. Besides the functional tests, several non-invasive biomarkers associated with epithelial cell damage, including intestinal fatty acid-binding protein (iFABP), citrulline, zonulin and claudins, can be proposed [10]. Moreover, some of the markers related to inflammation, such as alpha-1-antitrypsin, can be used to assess the state of intestinal integrity [13]. Apart from the non-invasive tests, the expression of TJ genes and proteins is commonly used to assess the quality of the gut barrier; however, due to the invasive character of sampling, these assessments are more commonly used in animal and in vitro studies.

The sensitivity of the microbiota to dietary components indicates that the modern model of nutrition may be one of the reasons for increased intestinal permeability because the so-called Western diet, rich in highly processed products, leads to dysbiosis, i.e., quantitative, qualitative and functional changes in the microbiota [1]. In turn, the poor microbiota is one of the “leaky gut”-associated observations [14]. The substances able to directly influence the intestinal microbiota can be called biotics. The family of biotics include probiotics, prebiotics, synbiotics and postbiotics. Modulation of microbiota composition by biotics appears to be an interesting concept to enhance the gut barrier, to treat or even prevent the onset or aggravation of chronic diseases.

Considering the importance of the gut barrier integrity and the potential connection between the gut microbiota and gut barrier, in this review, we summarized the impact of biotics on the gut barrier and its permeability. Thus, the aim of this review was to show changes in the intestinal barrier integrity as a result of pro, pre-, syn- and postbiotics supplementation. Interest in the regulation of the intestinal barrier has increased over the past years, and this review aims to assess the effectiveness of taking biotics in enhancing the intestinal barrier. Our review shows that despite numerous studies in this field, there is no clinical evidence yet for the effectiveness of such supplementation in humans. We presented the current data and underlined the need for further studies to understand the mechanisms of microbiota modifications regarding gut barrier functionality.

## 2. Methodology

A literature search on the Web of Knowledge research platform was performed, searching all databases using the term “probiotic”, “prebiotic”, “synbiotic”, “postbiotic” AND “gut permeability”, “intestinal permeability”, “gut barrier”, “intestinal barrier”, in the topic and title to identify articles published up until 2022. To be included, studies had to meet the following criteria: (1) randomized clinical trials aimed to evaluate the effect of biotics on the gut barrier integrity; and (2) in vitro and in vivo studies evaluating the effect of biotics on the gut barrier integrity. Studies about the effect of biotics on the gut microbiota without the direct analysis on any parameters related to the intestinal barrier were excluded.

## 3. Probiotics

The initiator of the probiotic idea is Elie Metchnikoff, who observed that lactic acid bacteria (LAB) affect health and longevity. In addition, he proposed a concept for the modification of microbiota with fermented milk in order to eliminate the proteolytic bacteria responsible for the production of ammonium, phenol and indole (protein degradation products) [15]. The definition of a probiotic evolved over the years. The most up-to-date concept of the International Scientific Association for Probiotics and Prebiotics (ISAPP) was formulated in 2014. According to this statement, probiotics are “live microorganisms which when administered in adequate amounts confer a health benefit on the host” [16]. For the microorganism to be called a probiotic, it must meet several strict criteria. First of all, it should be emphasized that probiotics are living microorganisms [17]. Food products and dietary supplements referred to as “probiotics” very often do not meet this criterion, specifying the total number of microorganisms without distinguishing them into live and dead ones. Moreover, this value often refers to the number of bacteria in the product at the production time and does not refer to the entire shelf life for consumption [18]. Secondly, probiotics must be carefully characterized, and their properties are assigned to a given strain and not to the entire genus or species [19]. For this reason, the wording “natural probiotics” used for fermented products is incorrect because the vast majority of fermented food is obtained as a result of the activity of indigenous microorganisms, naturally occurring on the raw material, and not with the participation of probiotic strains with a confirmed status [20,21]. A properly characterized probiotic has to have a three-part definer, letter-numeric name, including the name of the species, genus and strain symbol [22]. Thirdly, probiotic strains must be safe to use [23]. Strain safety is determined by historical strain monitoring and updated scientific reports. The safety status is issued by appropriate institutions. In Europe, regulations in this area are issued by the European Food Safety Authority (EFSA). The EFSA has compiled a list of microorganisms that are safe for human consumption below the “Qualified Presumption of Safety” (QPS). The idea of QPS has some limitations. First of all, it is based on the thresholds for the healthy part of the population, and it is based on microorganisms that were referred to the EFSA before being placed on the European market [24]. Other countries have different safety criteria. For instance, the Food and Drug Administration (FDA) regulates this area in the United States. According to its regime, the safety of probiotics is referred to as generally recognized as safe, and the most commercially available probiotics, LAB, *Bifidobacterium* and yeast, have GRAS status [25]. Lastly, probiotics must have a proven health-promoting effect. The health benefits of a strain must be supported by at least one clinical trial conducted according to scientific standards [24].

In general, probiotics can be delivered as dietary supplements and fermented products containing strains with proven probiotic status. Dietary supplements contain carefully selected strain or strain compositions showing synergistic action [26]. Among fermented foods, probiotic foods are those enriched with a probiotic strain or resulting from the use of starter cultures that are a confirmed probiotic strain. Nevertheless, the probiotic potential of fermented foods prompted us to consider their role in maintaining the intestinal barrier.

Despite the stringent criteria set for probiotics, there is no shortage of evidence that probiotic products are adulterated. The distortions mainly concern the presence or the declared number of probiotic strains in a given dietary supplement or functional product. In order to meet the distribution of counterfeit products, in 2022, an innovative tool to detect this type of irregularity was developed [27]. The probiotic identity card (PIC) relies on the collaborative action of shotgun metagenomic sequencing and the enumeration of bacterial cells using flow cytometry. Regarding data, PIC is used to evaluate the quality of 12 commercial probiotic preparations, showing some inconsistencies in the viability and quantity of probiotic strains. This tool seems to be promising in the era of growing interest in probiotic foods and supplements. This will ensure the quality of probiotic products and allow you to actually use the health-promoting properties of probiotics.

**Figure 2:**
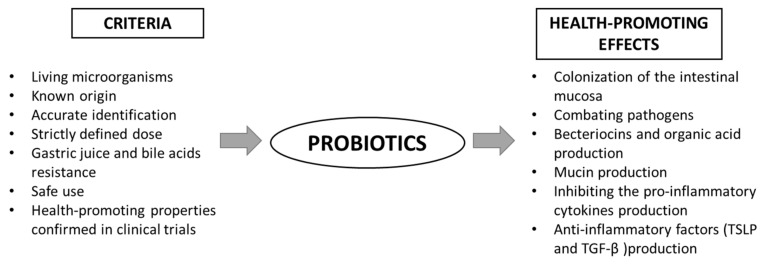
Scheme of the criteria for probiotics and their health-promoting properties. TGF-β—transforming growth factor β; TSLP—thymic stromal lymphopoietin.

The main benefits of using probiotics are: (I) the production of substances with antimicrobial properties, (II) competition with pathogens for adhesion sites on the intestinal epithelium, (III) immunomodulation and (IV) inhibition of toxin production by other bacteria [26]. In order to qualify a strain as a probiotic, it must meet certain health-beneficial criteria (Figure 2). The action of probiotics is varied and multidirectional. Among the health-promoting effects of probiotic activity, the most frequently mentioned are: immunomodulating properties, the support of the cardiovascular system, strengthening the gut barrier and modulating the intestinal microbiota [25]. However, the mechanisms of action in all of these contexts are not fully understood. The following paragraph presents the overview of recent data on the role of probiotics in gut barrier maintenance. However, it should be emphasized that despite the well-characterized, easily available, widely-studied probiotics and their proven pro-health effects, only a few are evaluated in terms of their efficacy in targeting the intestinal barrier. Therefore, in this review, we present the groups of probiotics and the results of studies closely related to the regulation of the intestinal barrier function.

### 3.1. Lactic Acid Bacteria

LABs are the largest group among probiotics. Although LABs are considered safe and have GRAS status, just a few of them have proven probiotic properties. Nevertheless, it is the most studied group of probiotics, also concerning gut barrier integrity.

The study by Anderson et al. [28] assessed the role of the *Lactobacillus plantarum* MB452 (according to new taxonomic classification [29,30]: *Lactiplantibacillus plantarum*) in the regulation of gene expression related to TJ formation. The study was conducted using an in vitro model with a Caco-2 cell line. It was shown that in the presence of *L. plantarum*, the trans-epithelial electrical resistance (TEER) was higher compared to the control. Interestingly, the increase in TEER was dose-dependent. The lowest dose (7 × 10^7^ CFU/mL) did not affect the TEER value, while the doses of 1.8 × 10^8^ and 3.0 × 10^8^ CFU/mL increased TEER by 15–20% and 42–51%, respectively. Moreover, the fluorescent analysis of immuno-stained TJs proteins, i.e., occludin, ZO-1, ZO-2 and cingulin, showed higher fluorescent signals than the control, thus pointing to the upregulation of these genes in the presence of *L. plantarum*. Additionally, the presence of probiotics altered the expression levels of occludin and its associated plaque proteins, tubulin and proteasome, possibly also affecting intestinal barrier function. The study by Yi et al. [31] also showed that *Lactobacillus reuteri* LR1 (according to new taxonomic classification: *Limosilactobacillus reuteri*) increased the expression of TJ genes. The study was performed in an in vitro model (IPEC-1) using ETEC K88 as a gut barrier damaging agent. The presence of *L. reuteri* LR1 (1 × 108 CFU for 6 h) was shown to reduce the adhesion and invasion of ETEC K88 as well as the permeability of the IPEC-1 cell monolayer induced by this pathogen. Additionally, the probiotic increased the expression of the TJ proteins ZO-1 and occludin, while it did not affect claudin-1 and F-actin. In the next stage of the research, ML-7 (a selective inhibitor of myosin light chain kinase, MLCK) was used and allowed them to conclude that the contribution of *L. reuterii* LR1 to increasing the content of ZO-1 and occludin is dependent on the MLCK pathway.

An interesting study was conducted by Montalto et al. [32]. The authors investigated the effect of *Lactobacillus acidophilus* (according to new taxonomic classification: *Lactobacillus acidophilus*) strain LB on TJ in aspirin-induced gut barrier permeability. In this in vivo study, the HT-29 cells were used as an intestinal epithelium model. The experiment was carried out in two variants, with aspirin and with aspirin in the presence of a probiotic. The results of the immunofluorescence analysis showed that the use of aspirin inhibited the expression of ZO-1 in the model intestinal epithelium, while the probiotic protected and alleviated the aspirin effects.

Blackwood et al. [33] evaluated the role of two *Lactobacillus* strains, *Lactobacillus rhamnosus* (according to new taxonomic classification: *Lacticaseibacillus rhamnosus*) and *Lactobacillus plantarum* (according to new taxonomic classification: *Lactiplantibacillus plantarum*) (10^7^ CFU/mL), in both in vitro and in vivo models including the Caco-2 intestinal epithelium model damaged using ethylene glycol-bis[beta-aminoethyl ether] tetraacetic acid (EGTA) calcium switch or lipopolysaccharides (LPS) and a rat model of intestinal barrier permeability. The studies were conducted in vitro (Caco-2) and in vivo (murine model). The results of in vitro studies indicated that *L. plantarum* (10^3^–10^5^ CFU/mL) protected the gut barrier and TJ proteins from damage caused by acetaldehyde (an ethanol metabolite) by acting on epidermal growth factor receptor (EGFR) signalling. A protective, EGFR-dependent effect was also observed in in vitro studies. Moreover, *L. plantarum* was shown to reduce the intestinal and liver damage induced by ethanol in a murine model. The presence of *L. plantarum* (10^6^ CFU/mL) inhibited the expression of pro-inflammatory cytokines (TNF-α, IL-1β, IL-6), monocyte chemotactic protein 1 (MCP1), the CXC motif chemokine ligand (CXCL) 1 and the CXCL2 genes in the colon.

In vivo studies using a murine model in the context of the interaction of *Lactobacillus* on TJ proteins were also proposed by Kaur et al. [34]. In this study, the effect of *L. acidophilus* (according to new taxonomic classification: *Lactobacillus acidophilus*) (10^6^ CFU/mL for one week) and epidermal growth factor (EGF) against gut barrier damage induced by *Clostridioides difficile* associated disease (CDAD) was evaluated. The mice were assigned to the following groups: receiving traditional drugs (ampicillin, cyclosporine and lansoprazole), drugs with probiotics and EGF and a group receiving probiotics after prior conventional treatment. Next, the presence of TJ proteins (ZO, occluding and α-actinin) was evaluated. It was shown that there were significantly fewer TJ proteins in the groups receiving drugs than in the other groups. Moreover, the highest amount of these proteins in the groups receiving the probiotic and EGF, previously treated with the therapeutic, was observed. This finding indicated the participation of probiotics and EGF in restoring the integrity of the intestinal barrier in mice.

An in vivo study with the use of broiler chickens was proposed by Nii et al. [35]. This study aimed to evaluate the effect of *Lactobacillus reuteri* (according to new taxonomic classification: *Limosilicabacillus reuteri*) on the mucosal barrier function of the gastrointestinal tract. Day-old chickens were given water (control group) or water containing 10^8^ CFU *L. reuteri* for 7 days, and then the duodenum, ileum and cecum were collected for histological and molecular examination. The markers of inflammation and intestinal barrier permeability were analyzed in the histological samples. The administration of *L. reuteri* increased intestinal villi height and crypt size. Moreover, oral administration of probiotics lowered claudin-6 and AvBD10 (avian β-defensin) and AvBD12 levels and increased JAM2 levels, indicating that this bacteria strengthens the intestinal barrier by regulating the expression of TJ proteins.

A study aimed to evaluate the effects of a probiotic on the gut barrier was conducted by Wang et al. [36]. The effect of *Lactobacillus casei* Zhang (according to new taxonomic classification: *Lacticasecibacillus casei*) (10^7^ CFU/g) on the intestinal barrier of piglets infected with ETEC K88 was investigated. The 14-day-old piglets were assigned to the control group or the experimental group in which the subgroups previously treated with *L. casei* or simultaneously with the ETEC pathogen were distinguished. The application of *L. casei* Zhang inhibited inflammation and prevented ETEC-induced intestinal damage. In addition, in animals receiving *L.casei,* the intestinal villi heigh increased and expression of TJ proteins (ZO-1 and occludins) were higher.

Francavilla et al. [37] conducted a randomized clinical trial (RCT) on the role of the probiotic strain *L. rhamnosus* GG (according to new taxonomic classification: *Lacticaseibacillus rhamnosus*) in children with irritable bowel syndrome (IBS) or functional pain. One hundred forty-one children were enrolled in this randomized, double-blind, placebo-controlled trial, lasting for eight weeks. An SAT was performed at the baseline and the end of the experiment. The group receiving the probiotic had a reduced intensity and frequency of abdominal pain, which was not observed in the placebo group. Notably, at the beginning of the study, 59% of children had elevated values of the intestinal permeability test, and the percentage of children with impaired gut barrier integrity decreased after the probiotic intake. This effect was not observed in the placebo group, which indicates that the administration of the probiotic *L. rhamnosus* GG had the ability to restore the integrity of the intestinal barrier in children with IBS. In another in vivo study, Mujagic et al. [38] evaluated the role of *L. plantarum* (according to new taxonomic classification: *Lactiplantibacillus plantarum*) WCFS1, CIP104448 and TIFN101 on gut permeability and mucosal gene transcription in healthy volunteers. In this study, indomethacin was used as the stress factor of the mucosal barrier. The seven-day intervention showed that *L. plantarum* TIFN101 most effectively influenced the regulation of genes related to intercellular adhesion and the synthesis of TJs and adhesive junction proteins (e.g., α-4 actinin, metalloproteinase-2).

### 3.2. Bifidobacterium

*Bifidobacterium* is the best-studied group of probiotics. There are many scientific reports on the health-promoting properties of these bacteria, and over 130 publications present the results of clinical trials using these bacteria [39]. The most studied strain representing *Bifidobacterium* is *Bifidobacterium animalis subsp. lactis*, also known as BB-12^®^. Many scientific reports indicate a relationship between *Bifidobacterium* strains and the enhancement of the intestinal barrier integrity.

The role of the *Bifidobacterium dentium* N8 on the intestinal barrier was investigated using Caco-2 as a model of the intestinal epithelium [40]. In this study, the permeability of the gut barrier was induced by LPS. The use of a probiotic strain increased the TEER and decreased the permeability of the Caco-2 epithelial model. Moreover, it was shown that the presence of *B. dentium* N8 increased the expression of ZO-1, occludin and claudin and decreased the expression of pro-inflammatory cytokines TNF-α, IL-1β and IL-6. Alleviating inflammation and enhancing the integrity gut barrier indicates that *B. dentium* N8 strain has the potential to support the therapy of inflammatory diseases.

In another in vitro study, the role of *Bifidobacterium*, *Enterococcus* and *Lactobacillus* strains on the regulation of the intestinal barrier was investigated using the Caco-2 model [41]. Based on the measurement of transepithelial resistance, it was found that *Lactobacillus* and *Bifidobacterium strains* counteracted TNF-induced intestinal permeability. *Bifidobacterium* strains were also shown to attenuate the damage to the Caco-2 cell monolayer. Moreover, the analysis of metabolites allowed to indicate acetate as an activator of strengthening the integrity of the gut barrier.

The results of the research obtained by Al-Sadi et al. [42] indicate that the *Bifidobacterium bifidum* (10^8^ CFU/mL) strain is an activator of the strengthening of the intestinal epithelial TJs. The study was performed in an in vitro model using Caco-2 as the intestinal epithelium counterpart. Strain screening identified BB1 as a potential therapeutic and prophylactic microorganism in leaky gut enteritis, thus highlighting the strain dependence of the observed effect. Further research focusing on the mechanism responsible for strengthening the intestinal barrier was carried out in vivo in a murine model. Strain BB1 was shown to be active by interacting with the Toll-like receptor 2 (TLR-2) located on the surface of the apical membrane. Moreover, activation of the p38 kinase pathway was shown to play a role in enhancing the intestinal barrier function.

The strain BB1 was shown to enhance gut barrier integrity in mice with necrotizing enterocolitis (NEC) [43]. The permeability of the intestinal barrier is related to NEC, but it is not known whether it is a cause or a consequence. The dose of *B. infantis* BB-02 probiotic was 3 × 10^6^ CFU in 20 μL. The intestinal permeability increased after 12 and 24 h in mice under NEC protocol. An increase in claudin-2 expression, a decrease in claudin-4 and claudin-7 and an increase in claudin-2 were observed after 6, 24 and 48 h, respectively. The results indicated that supplementation with *B. infantis* improves the intestinal barrier function and reduces the incidence of NEC. The NEC model of damage of the gut barrier was also used in another study assessing the usefulness of the *Bifidobacterium* strain in restoring gut barrier function in rats and using the LPS-induced epithelial permeability cell model (Caco-2) [44]. The presence of a probiotic strain increased the TEER and decreased the permeability in the Caco-2 monolayer. Moreover, the probiotic decreased the secretion of proinflammatory cytokines IL-6 and TNF-α and changed the release of ZO. Furthermore, the probiotic pretreatment increased the expression of occludin, claudin-3 and ZO-1 and did not disturb the protein localization within the TJs. An in vivo model showed that the probiotic supply reduced by more than half the incidence of NEC as well as contributed to the reduction of NEC symptoms. A reduction in the secretion of pro-inflammatory cytokines was demonstrated in vitro after the inclusion of the probiotic. Importantly, it was also shown that probiotics reduced the permeability of the gut barrier, reduced the level of zonulin in serum and increased the expression of TJ proteins as well as the normalization of their localization within the TJs.

### 3.3. Escherichia coli Nissle 1917

*Escherichia coli* Niessle (EcN) is one of the few Gram-negative bacteria with a confirmed probiotic status [45]. There are scientific reports showing the participation of EcN in relieving inflammation.

The disruption of TJs and an increase in intestinal permeability may result from the presence of enteropathogens [46]. The restoration of the intestinal barrier integrity by the probiotic EcN strain was studied, e.g., by combating enteropathogenic *E. coli* (EPEC). The study by Zyrek et al. [47] evaluated the influence of EcN on intestinal permeability caused by EPEC using an in vitro model of an epithelial cell monolayer represented by a polarized T84 cell line. The experiment was conducted in two variants—in coincubation with EPEC and after the EPEC infection. It was shown that the presence of a probiotic EcN strain restored the intestinal barrier in both variants, which was observed by altering the gene expression and redistribution of ZO-1 and specific protein kinase C (PKC) inhibitors. These findings somehow explain the mechanism of the reported therapeutic effect of EcN in IBD [48]. In turn, the study by Alvarez et al. [49] showed that the presence of EPEC in model epithelial cells (Caco-2 and T-84) resulted in the downregulation of TJ proteins (ZO-1, ZO-2, ZO-3, occludin and claudin). Moreover, the authors noted the change in the organization of the cytoskeleton by investigating the location of ZO-1 and F-actin. However, the outer membrane vesicles and soluble factors, which are products of the secretory activity of EcN and commensal ECOR63, were found to protect the intestinal epithelium from the damaging effects of the pathogen. Other microbes also contribute to the damage of the intestinal epithelium. Another example is *Campylobacter jejuni*, the most common foodborne pathogen [50]. The study using HT-29 as a model of the intestinal epithelium showed that *C. jejuni* reduced the expression of genes associated with intestinal cell permeability. The use of the EcN strain reduced the number of pathogen cells by approx. two logs, and the intracellular presence of *C. jejuni* was not demonstrated. Moreover, EcN induced the expression of genes related to the maintenance of the intestinal barrier function. Interestingly, the other probiotic strains used in the study (*Lactobacillus rhamnosus* GG, *Lactobacillus acidophilus* NCFM and *Bifidobacterium animalis subsp. Lactis*) showed no protective effect against *C. jejuni* invasion.

Another study using an in vitro model (HT-29/B6) aimed to investigate the mechanism by which EcN modulates the intestinal barrier function [51]. It was observed that the presence of EcN causes an increase in TEER and a decrease in mannitol permeability, which proves the strengthening of intestinal epithelial cells’ integrity and sealing the intestinal barrier. In a further stage of the research, the authors found that the effect was dependent on the TcpC protein of EcN. The induction of signalling pathways regulated by this protein leads to an upregulation of claudin-14, a protein that forms TJs of the intestinal epithelium.

In the study by Ukena et al. [52], EcN upregulated the expression of ZO-1 in gene and protein levels. This study was conducted in vivo and in a murine model. Firstly, the EcN was given to healthy mice, and an increase in the expression of ZO-1 and ZO-2 was observed. Then, the mice with DSS-induced colitis were supplemented with EcN and expression of ZO-1 increased. In addition, less weight loss in the body and a shortening of the colon under inflammatory conditions was observed. Moreover, in the model, when DSS and EcN were applied together, the probiotic had a protective effect on the intestinal integrity and increased the expression of ZO-1. Therefore, the study indicated that probiotic stain EcN influenced on intestinal barrier. Moreover, these findings suggested that EcN can be used as a preventive or therapeutic measure.

### 3.4. Bacillus

The genus *Bacillus* is spore-forming, Gram-positive bacteria with good probiotic characteristics [53]. These bacteria were also studied in the context of regulating the intestinal barrier function. For instance, Rhayat et al. [54] showed in an in vitro study (Caco-2) that *Bacillus subtilis* can regulate the intestinal barrier. In this study, the authors examined three strains, and one of them (Bs 29784) restored intestinal integrity and increased the expression of TJ proteins (occludin, claudin-2 and ZO-1). In another study, the impact of *B. subtilis* CW14 was examined against damage caused by ochratoxin A (OTA) in the Caco-2 model [55]. The use of OTA was shown to reduce the expression level of TJ proteins, while the presence of the *B. subtilis* strain reversed this effect by increasing the expression of ZO-1. The effect of *B. clausii* on the intestinal barrier was also demonstrated in the Caco-2 model with induced acute gastroenteritis (AGE, rotavirus infection) [56]. AGE occurs in children and manifests as diarrhea. Administration of *B. clausii* was shown to improve the health of children; however, the mechanism of action of the probiotic is unknown. This study tried to understand the mechanism of probiotic action. The probiotic administered at 3 × 10^8^ cells/mL to Caco-2 cells caused an increase in TEER that was lowered by AGE and increased expression of TJ proteins. Thus, this study suggested that the therapeutic effect of *B. clausii* in the treatment of AGE is associated with the improvement of the intestinal barrier function; however, it requires confirmation if further in vivo studies. The effect of probiotics belonging to *Bacillus* sp. on the intestinal barrier was also assessed by in vivo studies. For instance, Bao et al. examined *B. amyloliquefaciens* TL106 against damage of intestinal barrier caused by *E. coli* in a murine model [57]. Animals were supplemented with TL106 at 2 × 10^9^ CFU/mL per day for two weeks. The use of TL106 reversed the effects caused by *E. coli*. The intervention resulted in an increase in the body weight of the animals and a decrease in the level of pro-inflammatory cytokines (TNF-α, IL-1β, IL-6 and IL-8). In addition, an increase in the expression of TJ proteins (ZO-1, occludin and claudin-1) and the diversity of the gut microbiota was observed. Another study reported the impact of *B. cereus* HMPM18123 on the intestinal barrier in mice with DSS-induced colitis [58]. Probiotic administration increased the expression of TJ proteins as well as suppressed the TLR4-NF-κB-NLRP3 inflammasome signaling pathways. Moreover, the intestinal microbiota was rebalanced. Another study indicated the protective role of *B. licheniformis* CMCC 63516 on heat stroke in rats [59]. The *B. licheniformis* was introduced in quantity 10^8^ CFU/mL twice daily for 7 days. Administration of *B. licheniformis* reduced mortality, hypothermia, pro-inflammatory cytokines and multi-organ damage on induced HS. In addition, restoration of intestinal barrier integrity, expressed on the basis of permeability markers (D-Lactate and I-FABP and FITC-Dextran 4), was observed, and the diversity of the intestinal microbiota increased.

Stevens et al. [60] conducted the study in piglets and human subjects. In this study, the effect on the gut barrier and survival in gastrointestinal conditions of the carotenoid-producing *B. indicus* PD01 was examined. Pigs were fed a normal diet or a normal diet enriched with PD01 (2 × 10^9^ spores/kg of diet) for 23 days, and volunteers took a PD01 (5 × 10^9^ CFU per day) or a placebo (3 g maltodextrin) for three weeks. It was observed that the strains survived the gastrointestinal passage in both experimental systems. Higher TEER values and occludin expression levels were observed in the piglet group receiving the PD01 compared to the control. However, in the human trial, no effect of probiotics on SAT results was observed.

### 3.5. Clostridium (Clostridioides)

*Clostridioides* constitute a large group of commensal bacteria in the human digestive tract. The main role of *Clostridioides* is the production of butyrate, which plays a trophic function for enterocytes and enhances the integrality of the intestinal barrier [61].

There are some reports about the role of *Clostridioides* on the intestinal barrier examined by in vivo study. Liu et al. [62] evaluated the effect of *C. butyricum* in murine model DSS-induced colitis. The administration of *C. butyricum* in the amount 2 × 10^8^ CFU reduced the level of pro-inflammatory cytokines (TNF-α), IL-1β, IL-6, IL-10 and IL-13) and increased the integrity of the intestinal barrier, expressed based on the increased expression levels of TJ proteins and FITC-labeled 4-kDa dextran flux. Another study conducted in mice with DSS-induced colitis showed decreased levels of pro-inflammatory cytokines (IL-1β, TNF, and IL-13 and NF-κB) and increased expression of TJ proteins (claudin-1, claudin-2, occludin and ZO-1) after administration of *C. butryricum* in the amount of 10^8^ CFU for 7 days [63]. In another study, the impact of *C. butyricum* on the intestinal barrier was evaluated in piglets [64]. In this study, the effect of *Clostridoides* was examined against damage caused by enterotoxigenic *E. coli* K88. The *C. butyricum* administration (5 × 10^5^ CFU) was started on day 14 after contamination with *E. coli*. Supplementation with *C. butyricium* reduced the level of pro-inflammatory cytokines and increased the integrity of the intestinal barrier compared to the group not receiving the supplementation. Another study showed the role of *C. butyricum* in shaping the intestinal barrier function in broilers [65]. In this study, broilers were supplemented with *C. butyricum* in a dose of 1 × 10^9^ CFU/ kg) for 28 days. Among the evaluation of the influence of the intervention on caecal mucosa using high-throughput sequencing techniques and histological staining, the impact of *C. butyricum* on the expression of some genes associated with intestinal barrier function (claudins 2, 15, 19, and 23, TJ proteins 1, 2, and 3 and mucin 1) was also determined. It was shown that the intestinal barrier integrity parameters were improved after the intake of *C. butyricum*. In addition, dietary supplementation enhanced the antioxidant capacity, intestinal barrier function and stabilized the intestinal microbiota.

### 3.6. Enterococcus

The genus *Enterococcus* belongs to the LAB. However, considering that they are known as pathogens even more than commensals [66], we decided to discuss them separately.

Enterococci are characterized by high tolerance to changes in temperature and pH as well as the production of bacteriocins with antimicrobial activity (against pathogens and microorganisms that cause food spoilage) [67]. To date, Enterococci do not have GRAS status; nevertheless, many representatives are used as probiotics [66]. The impact of *E. faecium* NCIMB 10415 on intestinal integrity in pig (IPEC-J2) and human (Caco-2) intestinal epithelium models was evaluated in response to pathogenic *E. coli* treatment [68]. It was shown that the presence of probiotics increased TEER in the Caco-2 model. Moreover, no changes in TEER or mannitol flux were observed in both models after pre-incubation with the probiotic. The restorative effect of the probiotic on damage caused by the pathogenic strain of *E. coli* was observed in vitro in both cell types. Another study examined the effect of *E. faecium* WEFA23 against *Listeria monocytogenes* CMCC54007 challenge in vitro (Caco-2) and in vivo (murine model) [69]. An in vitro study showed that *E. faecium* restored damage to the intestinal barrier and decreased the level of pro-inflammatory cytokines. An in vivo assay showed that *E. faecium* caused an increase in body weight, decreased mortality as well as decreased cell count of *L. monocytogenes*. Moreover, the presence of *E. faecium* caused a reduction in fluorescein isothiocyanate (FITC)-dextran in serum (another substance used for absorption tests). The increase in intestinal barrier integrity was also observed on the basis of a higher level of TJ proteins (claudin-1, ccludin and ZO-1). In another study, He et al. [70] evaluated the potential therapeutic properties of *E. faecium* in mice with DSS-induced colitis. The supply of the strain in the amount of 10^9^ CFU per day reduced the level of pro-inflammatory cytokines (TNF-α, IL-1β, IL-6 and IL-10), restored the balance of microbiota and enhanced the intestinal barrier function. Intestinal permeability was assessed using SAT and measurement of the expression of TJ proteins. The expression of claudins and ZO-1 increased, and the injury of the intestines was repaired. Another study was performed in a broiler chickens model [71]. Wu et al. (2019) used *E. faecium* NCIMB 11181 in a dose 2 × 10^8^ CFU/kg in chickens with impaired intestinal permeability (necrotic enteritis, NE). The administration of the probiotic reversed the damage of the intestinal barrier caused by NE by increasing the expression of TJ proteins, mainly claudin-1.

### 3.7. Saccharomyces boulardii

*Saccharomyces boulardii* is a non-pathogenic yeast discovered in 1923. Over the years, it was studied and characterized, and it is successfully used as a probiotic in the prevention and treatment of gastrointestinal diseases. Due to the fact that *S. boulardii* is a yeast, unlike bacteria, it naturally has some characteristics of a probiotic, i.e., antibiotic resistance, tolerance of low pH and the presence of bile salts [72]. The role of *S. boulardii* in maintaining the intestinal barrier function is fairly well documented.

For instance, Dong et al. [73] examined the effect of *S. boulardii* on gut barrier function in DSS-induced colitis in a murine model. Mice were assigned to five groups: positive control, negative control, *S. boulardii* group, mesalazine group and mesalazine plus *S. boulardii* group. *S. boulardii* positively influenced the expression of ZO-1 and occludin and inhibited the secretion of TNF-α and IL-8. Moreover, yeast administration increased the amount of *Bacteroidetes* and decreased *Firmicutes*. Thus, *S. boulardii* was shown to reduce inflammation and protect the intestinal barrier in a murine model of colitis.

Li et al. [74] tried to elucidate the mechanism by which *S. boulardii* relieves IBD. In this study, mice received *S. boulardii* for 16 days before the DSS-induced colitis. *S. boulardii* was shown to reduce inflammation by reducing the secretion of pro-inflammatory cytokines. It was observed that yeast reduced colon damage, modulated microbiota composition and increased the production of short-chain fatty acids (SCFAs), which resulted in a protective effect in the course of colitis.

In the study by Justino et al. [75], the effect of *S. boulardii* CNCM I-745 on the Toll-like/MyD88/NF-κB/MAPK pathway activated during intestinal mucositis and in Caco-2 cells treated with 5-FU was assessed. The mice were divided into three groups: saline, saline and 5-fluorouracil and 5-fluorouracil with *S. boulardii*. The yeast was administered at 10^10^ CFU for a period of three days. In turn, in the in vitro model, cells were treated with 5-fluorouracil or 5-fluorouracil and LPS. Then, *S. boulardii* was used. In both variants of the experiment, the expression level of the inflammatory markers was assessed. It was shown that in both variants, *S. boulardii* reduced inflammation, which makes it a potential therapeutic solution in intestinal inflammation.

In the study by Terciolo et al. [72], the effect of *S. boulardii* CNCM I-745 on the integrity of the intestinal barrier was evaluated. Colon samples were collected from patients with IBD, chronic ulcerative colitis (UC) and Crohn’s disease (CD). Treatment of colon explants with the supernatant of *S. boulardii* was shown to protect epithelial cell morphology and maintain E-cadherin expression on the cell surface. Treatment of colon explants with the supernatant of *S. boulardii* was shown to protect epithelial cell morphology and maintain E-cadherin expression on the cell surface. As a consequence, the connections between enterocytes were restored.

Another study evaluated the role of multistrain probiotics in maintaining the intestinal barrier. Nébot-Vivinus et al. [76] investigated the role of Lactibiane Tolerance^®^ (LT) on the gut barrier in vitro (T84) and in vivo (mice). The study was conducted under several conditions: in vitro in basal and inflammation state (induced with LPS or with conditioned medium of colonic biopsies from IBS patients) and in vitro in visceral hypersensitivity state induced by chronic stress or by colonic perfusion of faecal supernatant from patients with IBS. In vitro, the probiotic enhanced the gut barriers when it was given under the inflammatory intestinal model. No effect in basal conditions was observed. Moreover, this effect was dose-dependent, and supplementation of 10^9^ LT increased gut integrity better than the dose of 10^6^ LT. In vivo in both experimental variant supplementation with LT increased gut integrity. Moreover, the increase in occludin expression was observed in vitro and in vivo. The results of this study indicated that LT showed therapeutic and not preventive effects in vitro and showed anti-visceral hypersensitivity action in vivo. Supplementation with two probiotic strains was used in the study by Chen et al. [77]. In this study, the intake of *Lactobacillus acidophilus* (according to new taxonomic classification: *Lactobacillus acidophilus*) and *Bacillus subtilis* was evaluated in relation to the intestinal barrier function and mucosal immunity in hens. The animals were divided into three groups: normal diet, normal diet with low-dose of both strains (250 mg/kg) and normal diet with high-dose of both strains (500 mg/kg). The results showed an improvement in the parameters of the intestinal barrier and mucosal immunity in the groups supplemented with probiotics, but the effects were stronger in animals receiving a higher dose of probiotics. A decrease in the level of pro-inflammatory cytokines (IL-6, TNF-α, and IFN-γ) was demonstrated in both groups receiving probiotics, while an increase in the expression of TJ proteins (occludin, claudin-1, and ZO-1) was observed in animals with high-dose of probiotics. Additionally, the supplementation of diet with probiotics increased the diversity of the intestinal microbiota and caused beneficial changes in the metabolism of amino acids and lipids.

The available scientific reports indicate a possible relationship between probiotics and the intestinal barrier. Most studies showed that probiotics could reduce inflammation and restore the integrity of the gut barrier. There are many reports showing the potential of probiotics and potentially probiotic strains in regulating intestinal barrier function; however, most of the research was carried out by in vitro or animal models. The clinical trials on probiotics and intestinal barriers are still limited. Therefore, there is a need for well-designed clinical trials to obtain information on the effects of probiotics on the human gut barrier.

### 3.8. Fermented Products

Fermented products are foodstuff produced with the participation of microorganisms. Autochthonous microorganisms present in raw material (fruits, vegetables, grains) or starter cultures shape the organoleptic profile of the final products [78]. According to the previously stated definition, probiotics are living microbial cells, so this basic criterion for probiotics is often not met in fermented products due to technological conditions in which bacteria do not survive [79,80]. Moreover, bacteria considered as probiotics are well characterized, and their characteristics are assigned to the strain. In the case of fermented foods, especially those prepared in a traditional way, even if the product contains live bacterial cells, the exact characteristics of the strains are usually not known [81]. Nevertheless, fermented foods have several health-promoting properties, including relieving inflammation, supporting the cardiovascular system or immunomodulating properties [82]. Bacteria present in fermented products are often referred to as potentially probiotic strains [83,84]. Therefore, microorganisms isolated from fermented foods are tested for probioticity [85]. One of the main criteria set for strains isolated from fermented foods is the capacity to survive in conditions of the gastrointestinal tract (tolerance to bile salts, acid, pepsin and pancreatin) was assessed [86].

There is a limited number of studies that evaluate the effect of fermented products on gut barrier integrity. For instance, Putt et al. [87] tried to explain how yoghurt affects the gut barrier. In this study, the authors used the Caco-2 cell model. Inflammation was induced by a “cocktail” of pro-inflammatory cytokines. Higher values of TEER were observed in the yogurt-treated model, indicating a lower permeability of the cell monolayer. Additionally, the expression levels of the TJ protein (claudin-1, ZO-1 and occludin) were higher in the yogurt-treated system than in the inflammatory model. These results indicated that yogurt might be a valuable product in maintaining intestinal barrier integrity, acting by regulating gene expression of the TJ proteins.

Another study examined the effect of fermented blueberry pomace on the gut barrier in a murine model [88]. The animals were divided into a group that was fed a normal diet and a high-fat diet with or without supplementation with fermented blueberry pomace. It was shown that enrichment of the diet with fermented blueberry pomace relieved inflammation and improved the morphology and integrity of the intestinal barrier, as evidenced by increased expression of ZO-1, claudin-1, claudin-4 and occludins. Moreover, an increase in the expression of NF-κB-MLCK was observed, which may indicate the involvement of this pathway in the regulation of intestinal barrier function by fermented blueberry pomace.

Probiotics and potentially probiotic strains were tested for their health benefits for years. The worldwide increase in leaky gut-related diseases forces the scientific community to seek preventive and therapeutic solutions to maintain the integrity of the intestinal barrier. Although fermented foods are believed to be health-promoting, it is difficult to demonstrate strong evidence of their efficacy. Fermented products are specific to a given geographical area (availability of raw material, culture, tradition), which makes it difficult to reproduce the scientific evidence. Moreover, the indigenous microbiota of individuals is highly variable, which also makes it difficult to draw definitive conclusions. Nevertheless, fermented foods improve the health parameters of the body, and it is worth including them in the diet, even if their health benefits result from other factors than the presence of (potentially) probiotic strains.

## 4. Prebiotics

**Figure 3:**
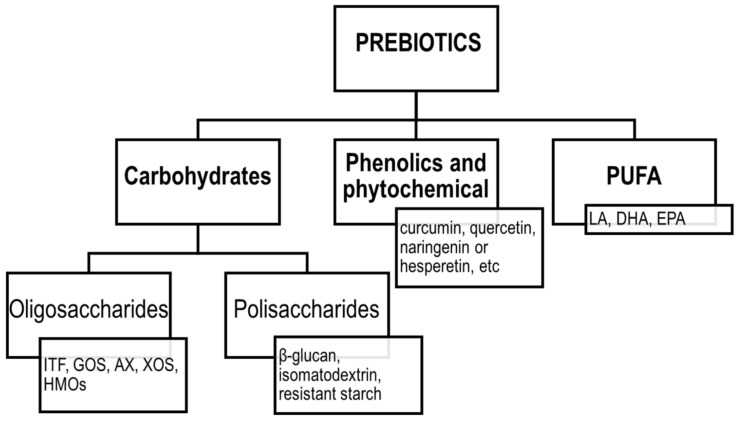
Classification of prebiotics according to ISAPP. ITF—inulin-type fructans; GOS—galactooligosaccharides; AX—arabinoxylans; XOS—xylooligosaccharides; HMOs—human milk oligosaccharides; LA—linoleic acid; DHA—docosahexaenoic acid; EPA—eicosapentaenoic acid; PUFA—polyunsaturated fatty acids.

The concept of prebiotics was proposed for the first time in 1995 by Gibson and Roberfroid [89]. Since then, the definition of prebiotics has evolved, and according to the ISAPP, the recent consensus definition implies that prebiotic is “a substrate that is selectively utilized by host microorganisms conferring a health benefit” [90]. Thus, the concept includes three essential parts: a substance, a physiologically beneficial effect and a microbiota-mediated mechanism. According to the original assumptions, the group of prebiotics included mainly fermentable carbohydrates, such as soluble fibres, inulin, fructooligosaccharides (FOS), galactooligosaccharides (GOS) and more recently, human milk oligosaccharides (HMOs). However, the current definition was extended to include also non-carbohydrate substances, such as polyphenols and polyunsaturated fatty acids (PUFA) [91]. The classification of prebiotics according to the new definition is presented in Figure 3.

The common characteristic for prebiotics is that they must confer a beneficial physiological effect on the host and that effect should derive at least in part from the utilization of the compound by resident microbes. To date, prebiotics are found to be very effective in the intestinal tract (mainly in the colon) by balancing or restoring gut microbiota both in the lumen and at the surface of the epithelium, with the special emphasis on the stimulation of growth of *Lactobacillus* and *Bifidobacterium* sp. [91]. Shifting the gut microbiota into a so-called healthier one may have immunomodulatory effects and can exhibit modulating, metabolic, trophic and protective mechanisms, such as the production of bacterial metabolites, including SCFA, which influence epithelial cell metabolism, turnover and apoptosis.

The positive effect of prebiotics on mineral absorption, bodyweight management and gut health modulation are repeatedly reported and summarized [91,92]. However, the effect of the prebiotic intake on the gut barrier is not fully articulated yet. The following paragraph presents the overview of studies focusing on the effect of individual prebiotics on the intestinal barrier function.

### 4.1. Inulin-Type Fructans

Inulin-type fructans (ITF) are water-soluble mixtures of oligo- or polysaccharides composed of β-D-fructosyl units linked by (2–1) glycosidic bonds [93]. Based on the degree of polymerization (DP), ITF can be further divided into long-chain inulin (DP > 10) and short-chain oligofructose, also called FOS (DP < 10) [94]. The changes in the gut microbiota caused by ITF are suggested to indirectly prevent gastrointestinal and systemic infections in both animal and human studies. The possible mechanism suggested to be responsible for the protective role of ITF is the bacterial antagonism and competition of so-called positive microbiota with pathogens as well as the trophic effects on the intestinal epithelium [92]. In vitro and animal studies show a great promise of the ITF intake on gut barrier integrity. However, there is no consensus with human studies to date.

A recent in vitro study was proposed to understand the mechanism of action of ITF on AMP-activated protein kinase (AMPK) and TJ assembly [95]. The authors investigated the effect of FOS under two conditions, inflammatory and non-inflammatory, using a human intestinal epithelial cell line derived from a colorectal adenocarcinoma patient (T84). It was reported that FOS in a dose of 0.1 mg/mL was capable of inducing AMPK phosphorylation via a calcium-sensing receptor (CaSR), independently from the gut microbiota. Moreover, FOS reversed the ability of LPS to suppress AMPK activity and TJ assembly through the same pathway [95]. A study with a porcine cell line was performed by Uerlings et al. [96]. The authors compared the effect of digested inulin (after the hydrolysis-dialysis steps) and fermented inulin on the expression of gut barrier genes. Digested inulin upregulated adherence and TJ gene expression levels (occludin, claudin-3 and ZO-1, while *CDH1*, mucin 1 (MUC1) and reduced the expression of claudin-3, which can suggest a beneficial impact of inulin directly on the gut epithelial barrier. Fermented inulin induced higher expression of *CDH1*, claudin-1, -3, epidermal growth factor receptor (EGFR) and MUC1 compared to digested inulin [96]. Such changes indicated that the fermentation step might endorse the production of beneficial metabolites, such as butyrate, which enhance the integrity of the gut barrier.

Several in vivo studies using various animal models aimed at evaluating and understanding the effect of ITF on the gut barrier were reported. Cani et al. [97]. hypothesized that prebiotic-induced modulation of gut microbiota normalizes intestinal permeability by a mechanism involving glucagon-like peptide-2 (GLP-2), consequently reducing inflammation and metabolic disorders during obesity and diabetes. To verify this assumption, they performed a series of studies with ob/ob mice. Mice treated with oligofructose exhibited lower plasma LPS and cytokines concentrations compared to the placebo group. Moreover, a decreased hepatic expression of inflammatory and oxidative stress markers was detected in the prebiotic group, which was associated with reduced intestinal permeability and improved integrity of TJs. Prebiotics also increased GLP-2 production, and the GLP-2-dependent mechanism was proposed to be responsible for improved gut barrier functions [97]. The effect of FOS on the functions of gut barrier in the methionine-choline-deficient mice with nonalcoholic steatohepatitis was reported by Matsumoto et al. [98]. In this study, only the mice supplemented with 5% FOS maintained a normal gut microbiome. Moreover, positive morphological changes were observed after FOS intake. Mice from the prebiotic group had significantly higher villus and longer small intestines compared to control mice. Additionally, the expression of TJ proteins examined via ZO-1 staining increased in mice fed with FOS, confirming the positive effect on the gut barrier [97]. FOS was also found to be a promising therapeutic and prophylactic agent in mucositis induced by 5-fluorouracyl in BALB/c mice [97]. In the study of Carvalho et al. [99], intestinal permeability, TJ expression (ZO-1 and occludin) and propionate concentration within physiologic levels remained unchanged despite the induction of mucositis. Moreover, in the groups fed with FOS, increased levels of butyrate and reduced bacterial translocation and inflammatory infiltrate were observed. Notably, treatment with FOS before and during the induction of the disease was found to be most efficient in maintaining histological and morphometric parameters as well as IgA production and protecting the gut barrier. On the other hand, the FOS did not induce mucus production, which was reduced in the animals with induced mucositis [99]. Beisner et al. [100] designed an in vivo study aimed to evaluate whether inulin can improve the gut barrier function damaged by the Western diet given to mice. The authors applied the Western diet enriched with 10% inulin in mice for 12 weeks. The supplementation with inulin induced expression of Paneth cell α-defensins and matrix metalloproteinase-7 (MMP7), which were reduced by the Western diet. Additionally, the intestinal permeability, measured using polyethylene glycol 4000 (PEG4000), was reduced in mice fed with inulin.

Yacon flour (YF) (*Smallanthus sonchifolius*), which is a source of FOS, was incorporated into the diet of rats with colon cancer by the Brazilian group [101,102]. In the first study [101], YF in a dose delivering 7.5% of FOS was added for eight weeks to the diet of rats with induced colon cancer. Rats with cancer presented significantly worsened parameters of the gut barrier compared to the healthy control. The animals fed with YF also had significantly reduced urinary excretions of mannitol and lactulose compared to the control group. The ratio of lactulose to mannitol change reduced by 40% after YF incorporation, suggesting the positive effect of prebiotics on the gut barrier. The second study by the same group confirmed these findings [102]. In this study, the dose of YF was reduced to provide 5% of FOS. After the intervention, the urinary excretion of lactulose was not affected; however, the improvement in mannitol excretion was noted. Consequently, the lactulose to mannitol ratio was reduced in the animals fed with YF, contrary to the rats without prebiotic.

The in vivo studies showed a great potential of application of ITF to restore the gut barrier. However, despite these promising results, and although ITF is one of the most studied prebiotics, there is a limited number of clinical studies evaluating its effect on the gut barrier. Olguin et al. [103] conducted a randomized, double-blind, controlled clinical trial in patients with burn injuries, supplemented with 6 g/day of oligofructose or placebo for 15 days. Gut permeability was evaluated by the sugar absorption test before and after the intervention. The ratio of lactulose to mannitol was significantly elevated in the burn patients, suggesting the impaired gut barrier. The sugar absorption test results improved progressively from day 1 to 21, irrespectively on the applied supplement, suggesting no effect of the prebiotic intake. However, as the author reported [103], the sucrose excretion was highly increased in the patients, suggesting the major defects of permeability in the upper gut, mainly at the gastric level. Therefore, it cannot be surprising that the prebiotic, which works mainly in the colon, did not alleviate the intestinal permeability in the upper intestinal tract.

Another study investigated the effect of the inulin-enriched pasta on the intestinal permeability measured by SAT as well as GLP-2 and zonulin concentrations in healthy young subjects [104]. Twenty young men were enrolled to consume pasta containing 11% of inulin or control pasta without prebiotics for five weeks. After the intervention, the lactulose recovery was lower in the inulin group, while there was no difference in the mannitol excretion. Consequently, the ratio of lactulose to mannitol was reduced in the individuals consuming prebiotics. Moreover, the inulin consumption also resulted in reduced zonulin and increased GLP-2 values. All the results indicate that the incorporation of inulin into the pasta has a beneficial effect on the gut barrier in healthy men [104]. These results are quite surprising considering that the study was performed in healthy subjects which did not have any gut problems and did not have impaired gut barriers. It is rather impossible that the barrier can be “more tight” than the perfectly physiological barrier.

A study on the effect of oligofructose-enriched inulin on the gut barrier in patients T1D was reported by Ho et al. [105]. The authors designed a randomized, placebo-controlled trial in children 8 to 17 years of age with T1D supplemented with prebiotic for 12 weeks. After three months, a significant increase in the relative abundance of *Bifidobacterium* in the prebiotic group was noted, which was no longer present after the 3-month washout. Moreover, a modest improvement in the intestinal permeability, measured by the sugar absorption test, was observed, accompanied by a higher content of C-peptide. After three-month prebiotic intake, a decrease in intestinal permeability was noted, while in the placebo group, an increase in their intestinal permeability was observed, although these differences did not reach statistical significance [105]. Importantly, at the baseline, only one-third of the patients had elevated values of the sugar absorption test.

Another study was conducted with subjects with at least one criterium for higher risk of type 2 diabetes (T2D). In this proof-of-concept study, 24 adults at risk of T2D were supplemented with 10 g/day of inulin or placebo for six weeks [106]. No effect of the prebiotic supplementation was observed in the intestinal permeability markers, plasma endotoxin concentration or LPS-binding protein concentration, despite a significant bifidogenic effect. It could be explained by the fact that the participants of this study were relatively healthy (i.e., did not have impaired glucose tolerance or confirmed T2D).

Celiac disease is suggested to belong to diseases characterized by the impaired gut barrier [107]. A randomized, placebo-controlled study was proposed to evaluate the effect of 12-week supplementation of a gluten-free diet (GFD) with oligofructose-enriched inulin (10 g per day) on intestinal permeability in children with celiac disease treated with a GFD. The integrity of the gut barrier was assessed by the analysis of the zonulin, intestinal fatty acid-binding protein, claudin-3, calprotectin and GLP-2, as well as SAT. The authors reported the lack of the substantial effect of prebiotic supplementation on the gut barrier. Although the intake of oligofructose-enriched inulin increased the excretion of mannitol, which may suggest an increase in the epithelial surface, the majority of children in this study seem to have normal values for intestinal permeability tests before the intervention. The inclusion criterium was that children were following a GFD for at least six months, which could be enough to restore the gut barrier [108]. Notably, for individuals with elevated values, improvement in calprotectin and SAT was observed after the prebiotic intake. Although the results seem to be promising, as the authors underlined, it is difficult to draw a conclusion based on the small number of individuals with an impaired gut barrier [108].

To summarize, the in vitro and in vivo studies showed great promise in the effects of ITF on the gut barrier, which was not fully confirmed in clinical trials. However, the majority of studies were performed with participants without the ongoing impairment in the gut barrier integrity. Therefore, there is a need to design studies with participants with elevated intestinal permeability to check whether the effects of ITF observed in animal studies can be translated into humans.

### 4.2. Galactooligosaccharides

Galactooligosaccharides (GOS) consist of β-linked galactose moieties with galactose or glucose at the reducing end. Known GOS contain β-(1→2), β-(1→3), β-(1→4), or β-(1→6) linked galactose moieties and usually have a DP of 3–8. GOS are produced from lactose by β-galactosidase in a kinetically controlled reaction between enzymatic hydrolysis and transgalactosylation [109]. GOS are not digested in the upper gut and exhibit a strong bifidogenic effect. Commonly, GOS are used as a substitute for HMOs in infant formulas [110,111]. Many studies showed that the intake of GOS can improve gut health [112] thus it can be assumed that GOS can also affect the gut barrier function.

Akbari et al. [113] compared the effect of different formulations of GOS (syrup and purified GOS) and DP on the epithelial integrity in an in vitro model of intestinal barrier dysfunction. Vivinal^®^ GOS syrup was found to be the most efficient in the protection of monolayer integrity and TJ reassembly. Notably, GOS syrup was rich in oligosaccharides with DP of 2–3 and comparing the obtained effects with ITF of different DP; it could be seen that smaller DP are of greater importance for the protection of barrier integrity than oligosaccharides of longer chains. The authors concluded that the microbiota-independent effect of oligosaccharides depends on the oligosaccharide structure, DP and concentration of each fraction [113].

Similarly to ITF, several in vivo studies were performed to evaluate the effect of GOS on the gut barrier using various animal models. Barrat et al. [114] conducted a study with neonatal rats fed with milk formula with or without the mixture of GOS and inulin (ratio 88:12) from the 7th to 20th day of life. Supplementation with prebiotic formula resulted in an increase in bacterial translocation at day 18, which was not observed anymore at day 40. However, the colonic permeability assessed by an Ussing chamber and the expression of TJ genes were not altered by the prebiotic intake. Contrary, the expression of ZO-1 was reduced by 40%, and trophic effects in cecal mucosa were observed in a GOS/inulin group. The explanation of the increased bacterial translocation is unclear, but one of the possible reasons could be the rapid fermentation of the prebiotics leading to the higher concentration of organic acids, which can impair the gut barrier. However, it was not confirmed in this study [114]. In another study, Zhong et al. [115] evaluated the effect of GOS supplementation in enteral nutrition in rats with induced acute severe pancreatitis. Pancreatitis was accompanied by intestinal barrier dysfunction. In the groups supplemented with GOS, the number of faecal bifidobacteria, sIgA level in intestinal mucus, intestinal occludin mRNA level at days 4 and 7 and the intensity of intestinal epithelial apoptosis at day 7 was significantly higher compared to the standard enteral formula. The authors reported that prebiotic intake could decrease the bacterial translocation in mesenteric lymph nodes and livers and alleviate pancreatic inflammation [115]. A study analyzing the effect of GOS on the gut barrier in rats with alcohol withdrawal syndrome was proposed by Yang et al. [116]. The chronic consumption of alcohol for five weeks increased the serum concentrations of diamine oxidase (DAO), D-lactate and LPS, indicating the impairment of the gut barrier. The withdrawal of alcohol decreased the concentrations of the gut barrier markers; however, the values were still higher than in control, healthy groups. The alcohol abstinence did not restore the villus length and crypt depth. The supplementation of the diet with GOS for three weeks ameliorated the gut barrier functions, and despite no effect in crypt morphology, the villus to crypt ratios increased significantly, suggesting that GOS can be applied as an auxiliary therapy for improvement of gut health after long-term alcohol consumption [116]. Another situation when the increase in intestinal permeability is observed is ageing. An interesting study was reported by Arnold et al. [117], who evaluated the effect of GOS in age-associated gut permeability. The authors confirmed that older mice had increased intestinal permeability compared to younger animals. However, GOS supplementation (71.8 g per kg) managed to alleviate these effects in old animals. Moreover, in old mice fed with GOS, an increase in mucus abundance was noted. Notably, the GOS intake had no effect in young mice, which is understandable since the animals had no gut barrier impairment [117].

Pigs provide a very good, clinically relevant model for studies on the human intestinal tract [118]. Therefore, several in vivo studies were performed using pigs, aimed to mimic the effects of prebiotics which then could be expected in humans. An example of such a study is the research of Alizadeh et al. [119]. The study was performed with piglets whose intestinal maturation is similar to the human neonates and infants. Animals were fed with GOS from 3 to 26 days, which resulted in an increase in colonic fermentation, decrease in pH and bifidogenic effect. The expression of the TJ genes was upregulated in the piglets fed with GOS as well as a higher level of defensin porcine β-defensin-2 in the colon, suggesting that the GOS-supplemented formula promotes the gut development and defence mechanism in the intestine in neonates.

The promising results obtained in vivo required confirmation in the randomized clinical trials. Pedersen et al. [120] applied GOS in a dose of 5.5 g per day for 12 weeks in 29 men with T2D. The prebiotic intake had no effect on the intestinal permeability estimated based on the urinary recovery of _51_Cr-EDTA, although a decreasing trend was observed. However, again, the number of participants with elevated permeability at the baseline was lower than expected (only 28%), which can explain the lack of significant differences. In another study, 5 g of GOS was consumed by 151 volunteers with obesity for three weeks [121]. The effect was compared to the probiotic strains *Bifidobacterium adolescentis* IVS-1 (autochthonous and selected via in vivo selection) and *Bifidobacterium lactis* BB-12 (commercial probiotic allochthonous to the human gut), used individually or as synbiotic combinations. None of the treatments had a significant effect on intestinal permeability. However, when the hyperpermeability was induced by a high dose of aspirin, GOS or probiotics intake improved the gut barrier tightness. Importantly, no synergistic effect was observed when GOS and probiotic were applied simultaneously.

### 4.3. Other Oligosaccharides and Prebiotics

Oligosaccharides are a big group of compounds differing in the nature of monomeric sugars. Apart from the above-mentioned ITF and GOS, there are many oligosaccharides that could potentially serve as prebiotics. The structure, characteristic and occurrence in nature was described elsewhere [122]. The examples of studies applying less popular oligosaccharides to improve the gut barrier function will be presented below.

Ducray et al. [123] evaluated the effect of *Saccharomyces cerevisiae* fermentate prebiotic in the protection of intestinal barrier integrity in rats during heat stress. Prebiotic was applied for two weeks before the exposition to heat. Heat stress resulted in inhibition of TJ proteins expression, a decrease of Paneth and goblet cells, a decrease of beneficial and increase of pathogenic bacteria. The intake of prebiotic before the heat treatment reduced the negative changes in the gut caused by heat, limiting the disruption of Paneth and goblet cells homeostasis and maintaining expression of TJ proteins [123]. Xylooligosaccharides (XOS) are sugar oligomers composed of xylose units linked by β-(1,4) bonds, with DP ranging from 2 to 10. The effect of XOS on the gut barrier, pancreatic islet and salivary gland inflammation in non-obese diabetic (NOD) mice were evaluated [124]. XOS supplementation delayed diabetes onset and decreased cellular infiltrations in their pancreatic islets and salivary glands. Importantly, XOS intake reduced gut permeability markers in the small and large intestines, which was accompanied by the upregulation of the mucus-related genes. The authors concluded that supplementation with XOS in early life could regulate the barrier function in a microbiota-independent manner [124].

HMOs play an important role in infant health and development; thus, in the last few decades, many studies were conducted to characterize the beneficial biological functions of HMOs and uncover the mechanistic pathways by which they are exerted [125]. This research was expanded to search for similar structures in other mammals. The widespread of the dairy industry has prompted studies into the therapeutic value of bovine milk oligosaccharides (BMOs) [126]. Hamilton et al. [127] verified the hypothesis that BMOs could prevent the deleterious effect of the high-fat diet on the gut microbiota and intestinal permeability and attenuate the development of the obese phenotype in mice. Mice were fed with a high-fat diet with or without BMO or inulin (6%/kg). BMO significantlyattenuated weight gain, decreased adiposity and decreased caloric intake. Notably, BMO and inulin intake abolished the increase in paracellular and transcellular permeability in the small and large intestines caused by the high-fat diet.

Another example of a prebiotic applied for the improvement of the gut barrier was isomaltodextrin (IMD) [128]. IMD was given to mice with low-grade chronic inflammation induced by LPS. Mice fed with IMD had decreased plasma concentrations of endotoxin, reduced macrophage infiltration into adipocytes, and increased expression of mucin 2, mucin 4 and the TJ protein claudin 4. However, the intestinal permeability measured by D-mannitol recovery was not affected by the oligosaccharide intake [128]. Gao et al. [129] evaluated the effect of *Lycium barbarum* polysaccharides (LBPs), alone or together with aerobic exercise, on the intestinal microbiota, gut barrier function and hepatic inflammation in rats with nonalcoholic fatty liver disease (NAFLD). Intake of LBP in a dose of 50 mg/kg for eight weeks together or without aerobic activity upregulated the expression of ZO-1 and occludin in the colon. Moreover, it reduced gut-derived LPS and hepatic LPS-binding proteins. The authors summarized that LBP could be a promising auxiliary therapy for NAFLD.

Less popular oligosaccharides and prebiotics were also applied in clinical trials. Westerbeck et al. [130] compared the efficacy of neutral GOS, FOS and acidic oligosaccharides (AOS) on intestinal permeability in 113 preterm infants. Prebiotics were added to the enteral formula between the 3rd and 30th days of life. At the baseline, the low birth weight was associated with increased lactulose to mannitol ratio. Irrespectively on the applied type of feeding (breastfeeding or the prebiotic-enriched enteral formulas), the lactulose to mannitol ratio decreased in the first week of life. Importantly, the lack of effect of prebiotics on intestinal permeability could be explained by high doses of antibiotics used in the intensive care unit (75% of all infants), which could inhibit the bifidogenic effect [131].

Another candidate for prebiotic are arabinoxylans (AX). The structure of AX consists of a backbone of β-(1,4)-linked xylose residues, which are substituted with arabinose residues on the C(O)-2 and/or C(O)-3 position. Salden et al. [131] evaluated if the supplementation of the diet with AX could reduce the intestinal permeability in individuals with obesity. The consumption of the AX for six weeks did not affect intestinal permeability analysed using a multi-sugar absorption test. Moreover, the expression of TJ proteins was not changed after AX intake. However, gene transcription of occludin was upregulated in the group supplemented with 7.5 g of AX, while the transcription of claudin-3 and claudin-4 were upregulated in the group consuming 15 g of AX [130]. Maybe a higher dosage and increase of the intervention period could give more promising results.

### 4.4. Beta Glucans

Βeta-glucans are soluble fibres derived from the cell walls of algae, bacteria, fungi, yeast and plants. Depending on the origin, they can have different types of glucan linkages. The structure and the effect of β-glucan supplementation on gut health were reviewed and summarized by Shoukat and Sorrentino [132].

The effect of yeast β-glucan on gut inflammation in dextran sulfate sodium (DSS)-induced colitis in mice was evaluated by Han et al. [133]. The intake of 5% of yeast β-glucan for seven days reduced clinical symptoms, inflammatory infiltrates and cell apoptosis in the colon epithelium. Moreover, the prebiotic administration ameliorated intestinal permeability and the structural integrity of TJs, which were highly impaired by DSS treatment. Mice fed with β-glucan increased the expression of the TJ genes up to 90% [133]. These results suggest that β-glucan could be beneficial in restoring the increased intestinal permeability in intestinal inflammatory diseases. Porcine models were used for the analysis of the effect of β-glucan on the gut barrier [134,135]. In the first study, barley-derived β-glucan was applied in different dosages in weaning pigs [134]. The high dose of β-glucan resulted in an increase in mannitol permeability and tissue conductance. Although it suggests that a high dose of β-glucan altered paracellular permeability, an increase in the epithelium surface area would also increase mannitol permeability. However, no differences in villus height, mucosal height, crypt depth or intestinal wet weight or length were noted. Therefore, it can be assumed that β-glucans exert effects on barrier function by altering the permeability of the transcellular pathway. In the second study, the influence of the β-glucan on nutrient composition and mucus permeability was evaluated [135]. In vitro digestion indicated that 90% of the β-glucan in the diet was released in the proximal small intestine. The intake of β-glucan for three days reduced the mucosa permeability to 100 nm latex beads and lipid.

The results of the effect of β-glucan on the gut barrier in clinical trials are in general contradictory to animal studies. On the one hand, Skouroliakou et al. [136] showed that barley-derived β-glucan-enriched snacks consumed for a month by healthy volunteers had no effect on intestinal permeability. However, this is another study with subjects without any gut barrier problems, and positive change cannot be expected. The reply for that was a series of studies reported by Mall et al. [137,138,139], who evaluated the effect of β-glucan on gut health in various groups with increased gut permeability. In the first study, the authors compared the efficacy of yeast-derived β-glucan and AX on restoring colonic hyperpermeability in elderly subjects with gastrointestinal symptoms [137]. No difference in intestinal permeability was detected between young subjects and the elderly group without GI symptoms based on the SAT. The effect of nondigestible polysaccharides was assessed ex vivo in Ussing chambers mounted with colonic biopsies. The elderly with gastrointestinal symptoms had higher intestinal permeability compared to the subjects without gastrointestinal manifestation. β-glucan significantly attenuated hyperpermeability in the elderly with GI symptoms but not in healthy controls. The possible explanation given by the authors for the increase in intestinal permeability was the potential contamination of β-glucan with LPS during production from yeast or the induction of the burst of reactive oxygen species caused by β-glucan [137]. At the same time, AX decreased paracellular and transcellular hyperpermeability across the colonic mucosa of healthy controls but did not attenuate transcellular permeability in the elderly with GI symptoms. This study showed that different prebiotics have different effects on the gut barrier, and the response is dependent on the initial state of the gut [137]. In another study in elderly subjects with permeability induced by anti-inflammatory drugs, Mall et al. [138] applied oat β-glucan and AX in a dose of 12 g per day for six weeks. At the baseline, the gastroduodenal permeability, small intestinal permeability and colonic permeability were elevated. The administration of prebiotics had no effect on gut barrier function. In the third study, Mall et al. [139] investigated whether β-glucan alleviate hyperpermeability in patients with CD. β-glucan significantly attenuated mast cell-induced paracellular hyperpermeability in both patients with CD and controls. However, transcellular hyperpermeability was only significantly attenuated in villus epithelium. Ussing chamber experiments further demonstrated that the inhibitory effect of β-glucan was more pronounced in follicle-associated epithelium compared to villus epithelium in patients with CD and control. The pronounced effect of β-glucan in follicle-associated epithelium-culture was confirmed by in vitro studies, demonstrating the beneficial effects of β-glucan on intestinal barrier function.

### 4.5. Polyphenols

Polyphenols are a large group of secondary metabolites characterized by multiple phenol units. These bioactive compounds are widely present in various crops, fruit and vegetables and are strong antioxidants, suggested to reduce the risk of noncommunicable diseases [140]. According to the new definition of prebiotics, polyphenols also belong to this group since microbiota metabolize polyphenols in the gut, and their derivatives confer health benefits. The number of studies performed on polyphenols increased dramatically in recent decades; however, it is a very big group of compounds in which activity can differ significantly between the classes. More studies were conducted by in vivo animal models, but still, some clinical trials were performed, and a few more were registered on clinicaltrail.gov [141]. These studies differ in terms of the type of animals or subject population, disease state, duration and dose and type of bioactive compounds. A recent review of Plamada and Vodnar [142] presents polyphenol-gut microbiota interactions. The effect of polyphenols on the gut barrier functions was summarized in an excellent review [140]; therefore, in this review, only a brief summary will be presented.

An interesting study was conducted by Song et al. [143], who evaluated the effects of grape-seed procyanidins in controlling post-weaning diarrhoea using a rat model. After supplementation of diet with 250 mg/kg of grape-seed procyanidins, improved growth performance and reduced diarrhoea occurrence were observed. As a possible explanation for these changes, the improvement of the intestinal barrier function was proposed. The intake of grapeseed procyanidins significantly reduced urinary lactulose to mannitol ratios of weaning rats compared with the control as well as upregulated the expression of the intestinal mucosal TJ proteins. Therefore, it was concluded that the improvement of the gut barrier was responsible for the reduction of diarrhoea in weaning pigs [143]. The comparison of the restoring effect of different polyphenols was reported by Shigeshiro et al. [144]. The mouse model with DSS-induced colon damage was applied to compare the effect of curcumin, quercetin, naringenin or hesperetin on the gut barrier TJs. It was found that all polyphenols partially restored the gut damage, but the level of changes was dependent on the polyphenol tested. The naringenin was found to be most efficient in intestinal barrier amelioration compared to other analysed polyphenols. In another study, chlorogenic acid was found to be very effective in restoring the gut barrier in rats with gut injury caused by endotoxin infusion [145]. Chlorogenic acid intake decreased the lactulose to mannitol ratio, reduced ileum pathological grade and oxidative stress in the gut. A recent study showed that kiwifruit polyphenol extract applied in a dose of 50 or 100 mg/kg is capable of inhibiting the impairment of the gut barrier caused by the high-fat diet [146]. Moreover, polyphenol-rich extract upregulated the expression of TJ proteins, claudin-1, occluding and ZO-1.

Considering the clinical trials, their number is not so big yet. The MaPLE project (microbiome manipulation through polyphenols for managing gut leakiness in the elderly) is an example of a complex approach aimed to verify if the modification of a diet of elderly people, increasing the intake of polyphenols can alter the microbial ecosystem to reduce the intestinal permeability [147]. The multidisciplinary approach designed in this project helps us to understand the possible mechanisms in the polyphenolsmicrobiota–gut barrier interactions. The project also includes the involvement of a metabolomic approach to understand the molecular pathways involved [148]. The initial results of this project showed that 700 mg of total polyphenols daily for 8 weeks could reduce the level of zonulin in the elderly population [149]. However, further studies are required to confirm the clinical utility of polyphenol supplementation in gut barrier protection.

### 4.6. Polyunsaturated Fatty Acids

Another new member of the prebiotic family is polyunsaturated fatty acids (PUFA). Although they were mentioned in the ISAPP definition as candidate prebiotics [16], there is still a lack of complete scientific evidence confirming that PUFA can be considered prebiotics [150]. Notably, with recent knowledge, not all PUFA can be considered prebiotics. Omega-3 fatty acids (eicosapentaenoic acid (EPA; C20:5), docosahexaenoic acid (DHA; C22:6) and omega-6 linoleic acid (LA; C18:2) were proposed as potential prebiotic candidates [150]. Since the effect of omega-3 fatty acids on the gut barrier was summarized in a recent review by Durkin et al. [151], in this review, only a few examples of clinical trials will be presented.

Van der Mewre et al. [152] evaluated the effect of long-chain PUFA supplementation of the gut integrity, cognitive development and growth in rural African infants. One hundred seventy-two children were supplemented with 200 mg DHA and 300 mg EPA or 2 mL olive oil for six months. Although the applied supplementation increased the level of circulating PUFA, no improvement in the gut integrity, growth and cognitive development was observed. In another study, the effect of PUFA on the zonulin level and LPS activity was measured in 200 pregnant women with overweight [153]. Moreover, a 2 × 2 factorial design was applied to evaluate the effect of PUFA and probiotics on intestinal permeability in pregnancy. The analysis showed that intestinal permeability increased with the progression of pregnancy in women with overweight and obesity and was reflected in LPS activity. The supplementation with PUFA, with or without prebiotic, did not affect the zonulin concentration and LPS activity.

The results of the clinical trials showed that despite the big enthusiasm of the anti-inflammatory and epithelium protective effect of PUFA obtained by in vitro and in vivo studies [154], the clinically relevant effect in humans is not easy to detect. Maybe more studies need to be performed to optimize a sufficient dose and type of PUFA, which can then ameliorate gut barrier impairment.

## 5. Synbiotics

Synbiotics are defined as a product that contains both probiotics and prebiotics in one formulation [155]. According to the newest definition of ISAPP, synbiotics are defined as “a mixture comprising live microorganisms and substrate(s) selectively utilized by host microorganisms that confers a health benefit on the host” [156]. Notably, when applied together, prebiotic and probiotic can exert synergistic or strain/prebiotic-specified independent properties.

Chiu et al. [155] studied if synbiotics can alleviate the alcoholic liver disease by altering the microbial ecosystem and consequently the gut barrier integrity. To do so, Wistar rats were fed a liquid diet containing alcohol with or without a symbiotic for 12 weeks. In fact, ethanol consumption increased intestinal permeability. Notably, synbiotic supplementation (FloraGuard^®^) attenuated the plasma endotoxin and protected the ethanol-induced hyperpermeability of the gut. This study showed that synbiotics could have not only an effect on the gut barrier but also exhibit hepatoprotective function [155]. The confirmation of the positive effect of the synbiotics was also confirmed by Trindade et al. [157]. The mice model with 5-fluorouracil induced mucositis was used to evaluate whether symbiotic containing *Lactobacillus paracasei*, *Lactobacillus rhamnosus*, *Lactobacillus acidophilus* and *Bifidobacterium lactis* together with FOS for 13 days. Mice fed with synbiotics displayed a significant recovery of lesions and the integrity of the gut barrier. The effect was much more prominent compared to prebiotic alone.

Several randomized clinical trials were designed to investigate the effect of synbiotics on intestinal permeability. Jain et al. [158] administered synbiotic preparation of *Lactobacillus acidophilus* La5, *Bifidobacterium lactis* Bb 12, *Streptococcus thermophilus*, *Lactobacillus bulgaricus* and oligofructose, 15 g/day in 90 critically ill patients. After a one-week intake of synbiotics, the abundance of potentially pathogenic bacteria decreased in the nasogastric aspirates of patients. However, the authors did not observe any differences in intestinal permeability, septic complications or mortality. Similarly, no clinically relevant effect was observed by West et al. [159], who applied Gut Balance^TM^ synbiotic, which contains *Lactobacillus paracasei* ssp *paracasei* (*L. casei* 431^®^), *Bifidobacterium animalis* ssp *lactis* (BB-12^®^), *Lactobacillus acidophilus* (LA-5^®^), *Lactobacillus rhamnosus* (LGG^®^), two prebiotics (raftiline and raftilose) and bovine whey derived lactoferrin and immunoglobulins with acacia gum for 21 days in healthy, physically active individuals. The authors observed no effect on the activity of gut microbiota and gut permeability. Importantly, the individuals recruited in this study were in a good fit, and the impairment of the gut barrier function was not expected. Furthermore, no effect of the synbiotics formulation was noted in a study in 50 biopsy-proven non-alcoholic steatohepatitis [160]. In this randomized clinical trial, patients received *Lactobacillus reuteri* with guar gum and inulin for three months, and their intestinal permeability was established based on the SAT and LPS level before and after the intervention. Although synbiotic intake caused a reduction in steatosis, body weight and BMI, there was no effect on the gut barrier. Similarly, no effect of the synbiotic containing 1.5 × 10^10^ CFU Ecologic^®^ 825 + 10 g FOS on the gut barrier was observed in healthy adults challenged with indomethacin in a study by Wilms et al. [161]. Indomethacin induced the gut barrier impairment, increasing the ratio of lactulose to mannitol. Notably, the synbiotic was not capable of restoring the induced damages. However, it has to be kept in mind that this intervention was run for only two weeks thus the positive changes could be seen after a longer intervention.

Contrary, Usami et al. [162] reported that preoperative synbiotics attenuated the decrease in the gut barrier integrity in patients who underwent hepatic surgery. In this study, the synbiotic formulation of *Bifidobacterium*, *Lactobacillus* and galactooligosaccharides was applied preoperatively for 14 days and after the surgery for 11 days. Importantly, the intestinal permeability was assessed only based on diamine oxidase activity without functional testing, thus it is rather hard to draw conclusions. Another study reporting that synbiotics could be beneficial in gut barrier restoring was reported by Horvath et al. [163], who investigated the effect of a six-month supplementation with multispecies synbiotics (i.e., a combination of probiotics and prebiotics) on, among others, the gut barrier in patients with diabesity. The authors noted a significant reduction in the circulating zonulin level, suggesting positive changes in the barrier integrity. Zonulin changes were also measured in another study in individuals with elevated body weight [164]. Sixty participants were supplemented with synbiotics for three months. In the synbiotic group, a reduction in zonulin level was observed, together with the increased variety of the gut bacteria. A recent study showed that NATUREN G^®^ synbiotic can reduce intestinal permeability in patients with chronic kidney disease [165]. Two-month intake of synbiotics resulted in positive changes in the intestine, including a reduction of abdominal pain, constipation syndrome and small intestinal permeability.

To date, the contradictory effects of synbiotic administration were reported. Importantly, many studies revealing no effect was conducted in subjects without leaky gut or the intervention were run for a short time. Interestingly, some studies showed the positive effect of probiotics and/or prebiotics but no synergistic effect. Considering the inconsistent conclusion drawn by different research groups, more carefully designed studies must be conducted to evaluate the clinical utility of synbiotic administration and select the groups of patients who will benefit the most.

## 6. Postbiotics

Postbiotics are a relatively new concept. In accordance with the latest recommendations of the ISAPP, postbiotics are defined as the “preparation of inanimate microorganisms and/or their components that confers a health benefit on the host” [166]. Postbiotics include SCFAs (butyric, propionic, acetic), teichoic acids, neuropeptides derived from peptidoglycan, exopolysaccharides, enzymes and cell walls fragments [167]. They are most often produced as a product of dietary fibre fermentation. To date, butyric acid seems to be the most studies postbiotic; hence, in this review, only the studies with butyrate will be presented.

There are many scientific reports on the pro-health properties of butyrate [168,169,170], there are also clinical trials [170]. Regarding the subject of our review, butyric acid also has a proven effect on the integrity of the intestinal barrier. A recent excellent review summarized the association between the butyrate, gut barrier and inflammatory diseases [171].

A recent study by Huang et al. [172] aimed to elucidate the role of SCFAs in maintaining intestinal barrier function. The study was performed by an in vitro model (Caco-2), and TNF-α/IFN-γ was used to induce inflammation. Butyrate, acetate, propionate and succinate were tested therapeutically in a pathological intestinal epithelium model. It was shown that when using butyrate, there was an increase in the TEER. Additionally, the application of TNF-α/IFN-γ increased the level of claudin-2 and decreased the level of claudin-3, while butyrate treatment abolished this effect. This study suggested that butyrate, as a representative of SCFAs, is involved in restoring intestinal barrier function. Moreover, it was noted that this effect is due to the regulation of claudin-2 expression. The obtained results may be helpful in the treatment of ailments related to the permeability of the intestinal barrier.

The vast majority of studies on the role of butyrate in alleviating inflammation and maintaining intestinal barrier function were carried out in vitro or in an animal model [173,174]. One clinical trial is currently underway, looking at the effects of fiber consumption on intestinal permeability (based on clinicaltrial.gov database). Given that fibre is a substrate for butyric acid production, it is expected that this study will provide insight into the role of butyrate in maintaining the intestinal barrier in humans.

Considering that postbiotic is a fairly new term, and its definition is quite dynamic, it can be expected that more research on the effect of substances classified as postbiotics on the gut barrier will be reported in future. Additionally, the increase in diseases correlated with gut permeability is directing future research to understand the role of postbiotics in regulating gut barrier function.

## 7. Summary and Future Perspectives

Knowledge of the activity of various biotics is becoming more extensive. Initially, it was believed that most of the pro-health functions of biotics were related to gut colonization and combating pathogens. However, over time, it was also shown that biotics devoid of live bacterial cells, such as prebiotics or postbiotics, are able to maintain a balance in the intestinal ecosystem. An increasing number of scientific reports indicate that the microbiota and general homeostasis in the intestinal environment are factors that guarantee health. Therefore, various strategies to maintain eubiosis are desirable. The modern lifestyle and model of nutrition lead rather to dysbiosis, which is one of the reasons for intestinal permeability. Increasing intestinal permeability causes chronic inflammation, which is one of the aetiological factors of inflammatory diseases, which presently constitute a global challenge. The so-called “leaky gut” is associated with diseases such as IBS, CD, celiac disease, chronic liver disease, diabetes, food allergies and sensitivities, polycystic ovary syndrome, depression and anxiety. The multiplicity and diversity of diseases associated with the increase in intestinal permeability indicate the need for strategies to restore gut balance and explain the mechanisms underlying the maintenance of the intestinal barrier function. In this context, the use of biotics seems to be promising. In both in vitro and in vivo experimental models, pro, pre-, syn- and postbiotics were shown to enhance the intestinal barrier. Most studies indicate the regulation of gene expression of TJ proteins as a potential mechanism of biotics action on the gut barrier. The positive effect of biotics was repeatedly reported by in vitro and in vivo models. However, it was rarely confirmed in human trials. Another problem underlined in this review is that many clinical trials were conducted with the subjects without ongoing leaky gut; thus, improvement cannot be expected. Therefore, there is a need for well-designed clinical trials clearly demonstrating the role of biotics in restoring the integrity of the intestinal barrier. The available clinical trials on biotics have little focus on gut barrier function but rather on systemic effects. Therefore, further clinical trials are needed to show the relationship between biotics and gut barrier function in human subjects. Moreover, there is a need to explore whether alternative biotics, such as postbiotics, can affect the gut barrier. It is also necessary to understand the mechanisms that are regulated by various biotics for restoring intestinal integrity, which will allow for the development of therapies eliminating the causes of diseases associated with the leaky gut rather than just symptomatic treatment.

## Data Availability

Not applicable.
